# The prognostic factor for recurrence in advanced-stage high-grade serous ovarian cancer after complete clinical remission: a nested case-control study

**DOI:** 10.1186/s13048-021-00908-8

**Published:** 2021-12-20

**Authors:** Qiao Wang, Ying Zheng, Ping Wang, Jiawen Zhang, Hui Liu, Qingli Li, Rutie Yin, Ce Bian, Hongling Peng, Zhilan Peng

**Affiliations:** 1 Department of Gynecology and Obstetrics, Development and Related Diseases of Women and Children Key Laboratory of Sichuan Province, Key Laboratory of Birth Defects and Related Diseases of Women and Children, Ministry of Education, West China Second Hospital, Sichuan University, Chengdu, Sichuan 610041 People’s Republic of China; 2 Department of Gynecology and Obstetrics, West China Second Hospital, Sichuan University, Chengdu, Sichuan 610041 People’s Republic of China

**Keywords:** High-grade serous ovarian cancer, Advanced stage, Recurrence, cancer antigen 125, Prognostic factor

## Abstract

**Background:**

Women with advanced-stage high-grade serous ovarian cancer (HGSOC) are likely to have a bad prognosis. Relapses are common in patients even with no evidence of disease after primary treatment. We aimed to identify the prognostic factors for disease recurrence in these patients.

**Methods:**

A nested case-control study was conducted in a large medical center in Southwest China. The primary outcome was recurrence of disease within 3 years after clinical remission (CR). Cox regression was used to calculate the time to event analysis in different groups.

**Results:**

Ninety-seven patients were finally included. Fifty-seven patients (58.8%) relapsed within 3 years after CR. Among all the variables, the difference in posttreatment CA-125 level was statistically significant (*P* <0.05) between the recurrent group and the progression-free group in both univariate and multivariable analysis. A cutoff value was set at the median level in the recurrent group (10 U/ml) to categorize patients into two arms. In Cox regression, the posttreatment CA-125 level was identified as a prognostic factor for recurrence with an OR of 1.05 (95% CI: 1.02–1.10, *P* = 0.033). The median time (from initiation of treatment) until relapse was 25 months for patients whose posttreatment CA-125 levels were higher than 10 U/ml, while it was undefined for patients whose posttreatment CA-125 level were ≤ 10 U/ml. Patients with a higher posttreatment CA-125 level showed an increased risk for OC relapse compared to those with a lower posttreatment CA-125 level. Furthermore, as shown in line graphs recording serum CA-125 levels during follow-up in each recurrent case, the increments of serum CA-125 levels were delayed in recurrent OC patients who had a posttreatment CA125 level ≤ 10 U/ml compared with those with a higher CA-125 level.

**Conclusion:**

A low serum CA-125 level after primary treatment was a potential prognostic factor in women with advanced HGSOC.

## Introduction

Ovarian cancer remains one of the Top 10 most common cancers for global women in global cancer statistics 2020, and is the leading cause of death among all gynecologic malignancy [1]. Epithelial ovarian cancer (OC) comprises about 90% of malignant ovarian neoplasms. Due to the lack of specific symptoms at early stage, more than 70% of OC patients are diagnosed at advanced stage with a low 5-year overall survival rate [2]. The standard-of-care treatment for a newly diagnosed OC patient is debulking surgery combined with perioperative platinum-based chemotherapy [3]. Unfortunately, over two-thirds of advanced-stage OC patients might relapse within the subsequent 3 years after primary treatment [4]. Recurrent OC is generally incurable. Currently, the well-known factors with prognostic value for disease recurrence in OC patients included International Federation of Gynecology and Obstetrics (FIGO) staging, pathological type, outcome of debulking surgery, and platinum sensitivity [5]. Achieving optimal cytoreduction (with residual disease less than 1 cm in maximum diameter or thickness after debulking surgery) is one of the most important components to improve outcomes in advanced-stage patients. However, relapses are still common in patients even with no evidence of disease after primary treatment. Woman with high-grade serous ovarian cancer (HGSOC), which is the most aggressive histologic subtype and accounts for the majority of advanced-stage diseases, is more likely to have a bad prognosis [6]. In order to identify more valuable prognostic factors for disease recurrence, we conducted this nested case-control study to evaluate further characteristics of relapsed HGSOC patients with advanced disease who had achieved complete clinical remission (CR) after primary treatment.

## Methods

### Subjects

We reviewed the clinical characteristics and outcomes of patients diagnosed with advanced-stage HGSOC who were treated in West China Second Hospital, Sichuan University, People’s Republic of China, from January 2013 to December 2017. Signed informed consent forms were obtained from all patients whose clinical data were collected.

### Inclusion and exclusion criteria

Our study included OC patients who met all of the following criteria: (1) whose histologic subtype was high-grade (grade 2 or 3) serous ovarian cancer; (2) who was staged at an advanced disease (FIGO stage IIB to IVA); (3) who received a debulking surgery (through an open laparotomy) performed by experienced gynecologic oncologists with qualification for OC surgery; (4) who achieved optimal cytoreduction after debulking surgery; (5) who received postoperative chemotherapy with recommended regimens, such as paclitaxel (135–175 mg/m^2^, intravenously) followed by carboplatin (300–400 mg/m^2^, intravenously) or paclitaxel (135–175 mg/m^2^, intravenously) followed by cisplatin (75–100 mg/m^2^, intraperitoneally) for 6–8 cycles; (6) who achieved CR after primary treatment (optimal cytoreduction with fist-line postoperative chemotherapy); and (7) who had complete clinical data during follow-up after the primary treatment. CR, partial response (PR), stable disease (SD), and progressive disease (PD) were defined using Response Evaluation Criteria in Solid Tumor (RECIST 1.1) and evaluated by chest/abdominal/pelvic computed tomography (CT) scans and serum cancer antigen 125 (CA-125) level.

We excluded patients: (1) who was diagnosed with other histologic subtype or at an early stage; (2) who underwent debulking surgery with minimally invasive techniques; (3) who had residual disease ≥1 cm after debulking surgery; (4) who did not complete the postoperative chemotherapy or was resistant to platinum-based chemotherapy; (6) who had a PR, SD, or even PD at the end of primary treatment; (7) who had missing information during follow-up; or (8) who had comorbidities (e.g., severe cardiac, hepatic, renal, or blood system diseases) or was not a good candidate for systematic treatment of OC.

### Study design

This was a retrospective study, designed as a nested case-control study to evaluate the prognostic factors in the risk of OC relapse. The preexisting cohort included advanced-stage epithelial OC patients who had achieved CR after successful primary treatment in our hospital during the study period. Medical records of each participant were carefully reviewed. The information of eligible patients who met the inclusion criteria was collected from the hospital electronic medical database. The primary outcome in our study was clinical recurrence of disease within 3 years after CR. Recurrent disease was identified by symptoms, signs, imaging (chest/abdominal/pelvic CT, magnetic resonance imaging or fluorodeoxyglucose-positron emission/CT scans) and elevated serum CA-125 levels during follow-up. The recurrent group included cases who had a progression of disease within 3 years after primary treatment. Progression of disease was defined using RECIST 1.1. The control group included patients in the same cohort who had a disease-free survival more than 3 years.

The following patient characteristics were evaluated as variables in the analyses: age, body mass index (BMI), family history of cancer, serum CA-125 level, serum Human Epididymis Protein 4 (HE-4) level, FIGO stage, sign of pleural/ascitic fluid, diagnostic approach, the application of neoadjuvant chemotherapy (NACT) or primary debulking surgery (PDS), the outcome of primary surgery treatment, the amount and area of residual disease after primary surgery, the identification of lymph node metastasis, the regimen and number of cycles of chemotherapy, and the application of postremission therapy.

Other clinical outcomes, including duration of overall response (DOR), progression-free survival (PFS), and adverse events (e.g., myelosuppression, postoperative lymphocyst, deep venous thrombosis, or ileus), were further evaluated in the subgroup analysis.

### Statistical analysis

The statistical analyses were performed using the software of IBM SPSS Statistics 23.0. Data were appropriately analyzed by parametric tests or non-parametric tests. Continuous variables were reported as mean ± standard deviation (SD) and compared by independent-sample *t* tests between two groups. Categorical variables were reported as number (n, %) and compared by chi-square (*x*^*2*^*)* tests between two groups. A *P* value < 0.05 (two-sided) was considered statistically significant. Confounding factors were adjusted by subgroup analysis. Cox regression was used to calculate the time to event (PFS or DOR) analysis. Odds radio (OR) and 95% confidence interval (CI) of each variable were calculated to evaluate the potential prognostic factors of OC relapse. Survival curve in each subgroup was analyzed using the software of GraphPad Prism 8.3.0 and compared by Log-rank test.

## Results

Of the 150 patients in the advanced-stage epithelial OC preexisting cohort, 105 patients met eligibility criteria in this study. Eight patients (7.6%) were finally excluded because of the missing information. The characteristics of the included 97 patients were summarized in Table 1. The mean follow-up was 4.6 ± 1.5 years. Fifty-seven patients (58.8%) relapsed within 3 years after primary treatment. The median PFS and DOR in the recurrent group were 26.0 ± 8.1 months and 17.0 ± 8.0 months, respectively. There was no significant difference in age, BMI, family history of cancer, FIGO stage, sign of pleural/ascitic fluid, diagnostic approach, outcome of primary surgery, lymph node metastasis, regimen/number of cycles of chemotherapy, posttreatment serum HE4 level (measured at the end of primary treatment), or application of postremission treatment between the recurrent group and progression-free group (control group). However, we found the proportion of NACT with interval debulking surgery (IDS) and the posttreatment CA-125 level were higher in recurrent patients than those in control group (P <0.05). Furthermore, we conducted a subgroup analysis stratified by the application of NACT (Table 2). The difference in the posttreatment CA-125 level between recurrent group and progression-free group was statistically significant in the PDS subgroup, as well as in the IDS subgroup (*P* <0.05).

**Table 1:** Patient characteristics and univariate analysis between the recurrent group and the progression-free group

		All	Progression-free group	Recurrent group	***P***
		***n*** = 97	***n*** = 40	***n*** = 57	
**Age (years)**		51.1 ± 7.8	52.0 ± 7.9	50.5 ± 7.8	0.349
**BMI**		22.4 ± 3.0	22.7 ± 3.3	22.1 ± 2.8	0.292
**Family histroy**	**None**	72 (74.2%)	30 (75.0%)	42 (73.7%)	0.793
	**Other cancer**	21 (21.6%)	9 (22.5%)	12 (21.1%)	
	**Ovarian cancer**	4 (4.1%)	1 (2.5%)	3 (5.3%)	
**Serum CA-125 level (U/ml)**	**Pretreatment**	1374.9 ± 1653.8	1060.7 ± 1036.5	1595.9 ± 1955.8	0.093
	**Postprimary treatment**	8.8 ± 5.1	6.3 ± 2.2	10.5 ± 5.7	0.000
**Serum HE-4 level after primary treatment (pmol/l)**		60.9 ± 20.0	59.0 ± 20.1	62.1 ± 20.1	0.501
**Presence of pleural fluid (n, %)**		21 (21.6%)	8 (20.0%)	13 (22.8%)	0.806
**Presence of ascitic fluid ≥ 1000 ml (n, %)**		55 (56.7%)	26 (65.0%)	29 (50.9%)	0.213
**Stage (FIGO) (n, %)**	**IIB**	7 (7.2%)	4 (10.0%)	3 (5.3%)	0.562
	**IIIA**	6 (6.2%)	4 (10.0%)	2 (3.5%)	
	**IIIB**	8 (8.2%)	3 (7.5%)	5 (8.8%)	
	**IIIC**	72 (74.2%)	28 (70.0%)	44 (77.2%)	
	**IVA**	4 (4.1%)	1 (2.5%)	3 (5.3%)	
**Confirmaiton of diagnosis (n, %)**	**Preoperatively**	51 (52.6%)	17 (42.5%)	34 (59.6%)	0.208
	**Intraoperatively**	43 (44.3%)	22 (55.0%)	21 (36.8%)	
	**After incomplete surgery**	3 (3.1%)	1 (2.5%)	2 (3.5%)	
**Primary surgery (n, %)**	**IDS with NACT**	53 (54.6%)	16 (40.0%)	37 (64.9%)	0.022
	**PDS**	44 (45.4%)	24 (60.0%)	20 (35.1%)	
**Optimal cytoduction (n, %)**	**R0**	61 (62.9%)	24 (60.0%)	37 (64.9%)	0.673
	**R1**	36 (37.1%)	16 (40.0%)	20 (35.1%)	
**Areas with residual disease (n, %)**	**Pelvis**	13 (13.4%)	5 (12.5%)	8 (14.0%)	0.284
	**Upper obdomen**	6 (6.7%)	1 (2.5%)	5 (8.8%)	
	**Scattered milia**	17 (17.5%)	10 (25.0%)	7 (12.3%)	
**Systematic lymphadenectomy (n, %)**		74 (76.3%)	33 (82.5%)	41 (71.9%)	0.332
**Lymph node metastasis (n, %)**	**Not detected**	55 (56.7%)	24 (60.0%)	31 (54.4%)	0.662
	**Spread to pelvic lymph nodes**	24 (24.7%)	8 (20.0%)	16 (28.1%)	
	**Spread to para-aortic lymph nodes**	18 (18.6%)	8 (20.0%)	10 (17.5%)	
**Postoperative chemotherapy**	**Number of cycles**	7.2 ± 1.2	7.2 ± 0.9	7.2 ± 1.4	0.968
	**Paclitaxel/cisplatin (n, %)**	38 (39.2%)	15 (37.5%)	23 (40.1%)	0.501
	**Paclitaxel/carboplatin (n, %)**	29 (29.9%)	14 (35.0%)	15 (26.3%)	
	**Alternately (n, %)**	30 (30.9%)	11 (30.0%)	19 (33.3%)	
**Postremission therapy (n, %)**	**None**	71 (73.2%)	27 (67.5%)	44 (77.2%)	0.111
	**Chemotherapy**	22 (22.7%)	13 (32.5%)	9 (15.8%)	
	**Bevacizumab**	2 (2.1%)	0 (0%)	2 (3.5%)	
	**Pazopanib**	2 (2.1%)	0 (0%)	2 (3.5%)	

*BMI* body mass index, *HE-4* Human Epididymis Protein 4, *CA-125* cancer antigen 125, *FIGO* International Federation of Gynecology and Obstetrics, *NACT* neoadjuvant chemotherapy, *PDS* primary debulking surgery, *IDS* interval debulking surgery, *R0* no visible residual disease, *R1* residual disease less than 1 cm in maximum diameter or thickness

**Table 2:** Subgroup analysis stratified by the application of NACT between the recurrent group and the progression-free group

		PDS			IDS		
		Progression-free group	Recurrent group	***P***	Progression-free group	Recurrent group	***P***
		***n*** = 24	***n*** = 20		***n*** = 16	***n*** = 37	
**NACT**	**Number of cycles**				2.6 ± 0.5	2.6 ± 0.7	0.871
	**Paclitaxel/cisplatin (n, %)**				12 (75.0%)	28 (75.7%)	0.587
	**Paclitaxel/carboplatin (n, %)**				4 (25.0%)	7 (18.9%)	
	**Alternately (n, %)**				0 (0%)	2 (5.4%)	
**Serum CA-125 level after primary treatment (U/ml)**		6.2 ± 2.1	10.4 ± 4.6	0.000	6.6 ± 2.0	10.6 ± 6.4	0.001
**Adverse events (n, %)**	**Myelosuppression**	22 (91.7%)	13 (65.0%)	0.057	13 (81.3%)	28 (75.7%)	0.737
	**Lymphocyst**	4 (16.7%)	2 (10%)	0.673	5 (31.3%)	5 (13.5%)	0.148
	**DVT**	3 (12.5%)	1 (5.0%)	0.614	1 (6.3%)	4 (10.8%)	1.000
	**Ileus**	1 (4.2%)	0 (0%)	1.000	0 (0%)	3 (8.1%)	0.550

*CA-125* cancer antigen 125, *NACT* neoadjuvant chemotherapy, *PDS* primary debulking surgery, *IDS* interval debulking surgery, *DVT* deep venous thrombosis

**Fig. 1:**
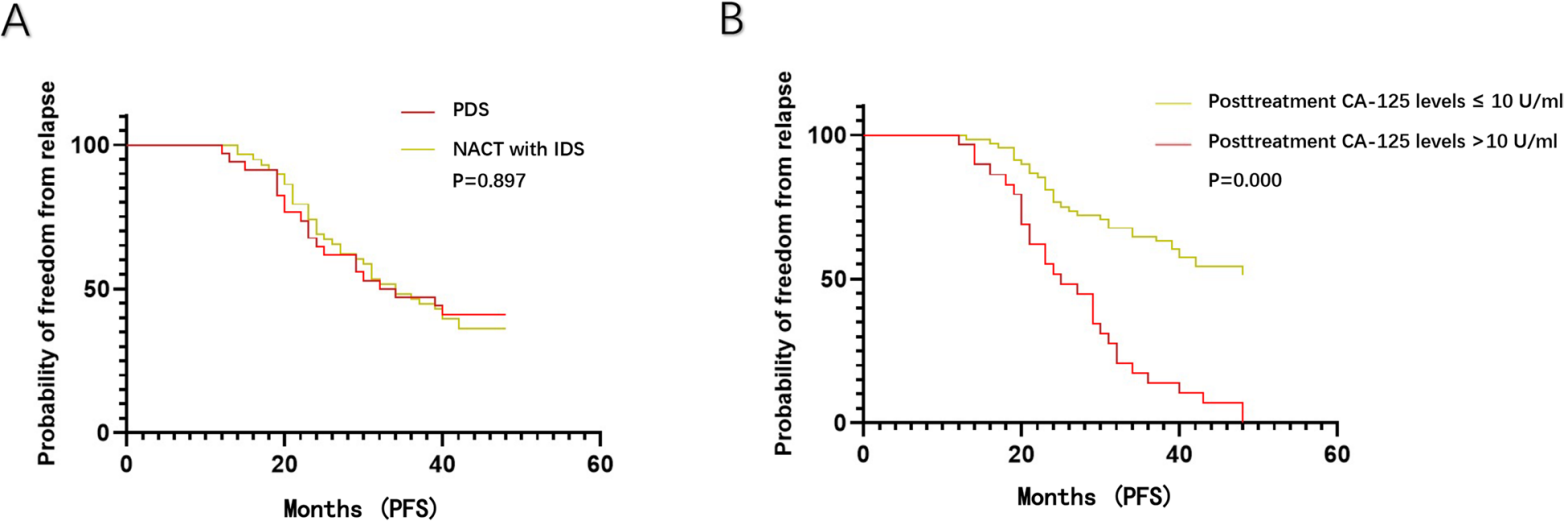
**A** Kaplan-Meier estimates of the rate of freedom from disease progression in the PDS group and the IDS group. **B**. Kaplan-Meier estimates of the rate of freedom from disease progression in patients with a posttreatment CA-125 level ≤ 10 U/ml and those with a posttreatment CA-125 level > 10 U/ml

**Fig. 2:**
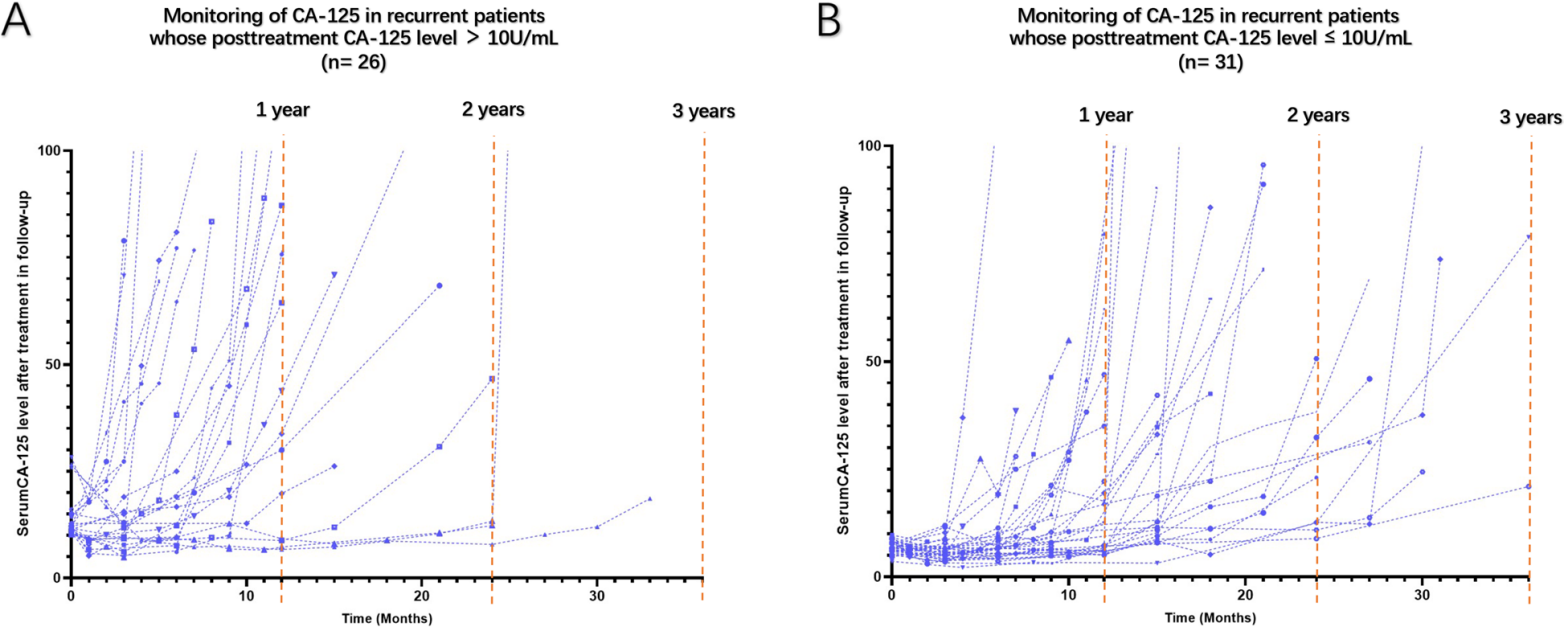
**A** The serum CA-125 levels recorded (after primary treatment) during follow-up in each recurrent OC patients with a posttreatment CA-125 level > 10 U/ml. **B** The serum CA-125 levels recorded during follow-up in each recurrent OC patients with a posttreatment CA-125 level ≤ 10 U/ml

In Cox regression (on the time to relapse), the posttreatment CA-125 level (measured at the end of primary treatment) was identified as a prognostic factor with an OR of 1.05 (95% CI: 1.02-1.10, *P* = 0.033), while the OR of the IDS was 0.93 (95% CI: 0.55-1.56, *P* = 0.782). The median time (from initiation of treatment) until relapse was 34.0 months for patients with PDS vs 33.0 months for those with IDS (*P*>0.05), indicating no significant difference in PFS between these two arms (Fig. 1A). As for the posttreatment CA-125 level, we set a cutoff value at the median level of the recurrent group (10 U/ml) to categorize participants into two arms. The median time until relapse was 25 months for patients whose posttreatment CA-125 levels were higher than 10 U/ml, while it was undefined for patients whose posttreatment CA-125 level were ≤ 10 U/ml. Moreover, patients with a posttreatment CA-125 level higher than 10 U/ml showed a statistically significant 266% increased risk for OC relapse compared to those with a posttreatment CA-125 level ≤ 10 U/ml (Fig. 1B). It indicated that patients with a higher posttreatment CA-125 level had worse prognosis with a significant shorter relapse-free survival. Furthermore, the changes in CA-125 monitoring during follow-up of recurrent cases were recorded as line graphs in Fig. 2. The elevations of serum CA-125 levels mostly happened within 12 months after primary treatment in recurrent OC patients with a posttreatment CA-125 level higher than 10 U/ml (Fig. 2A). As shown in Fig. 2B, the increments of serum CA-125 levels were delayed in recurrent OC patients who had a posttreatment CA125 level ≤ 10 U/ml (turning points apparently shifting to the right compared with those in Fig. 2A).

## Discussion

Our study showed that a lower serum CA-125 level at the end of primary treatment, as compared with a higher level, was a potential and good prognostic factor with regard to progression-free survival among women with advanced HGSOC.

It is well-known that serum cancer biomarker CA-125 is playing a very important role in detecting and guiding management of epithelial OC for the past three decades [7, 8]. CA-125 is also reported as a biomarker correlating with the therapeutic response and the monitoring of disease progression [9–11]. However, a more ideal biomarker should help assess the prognosis and make the most appropriate treatment strategy. Thus, it is reasonable to explore the prognostic value of serum CA-125 to improve individualized management of OC patients, especially for patients with an advanced disease and a high risk of relapse. Since pretreatment serum CA-125 levels usually correlate with the tumor stages and pathological types, it is hard to determine whether pretreatment CA-125 level is an independent risk factor of survival due to the confounding factors. In our present study, the subjects are patients with advanced-stage HGSOC. The strictly defined inclusion criteria limited the confounder in prognosis. There was no statistically significant difference in pretreatment CA-125 level between the recurrent group and control group, either in univariate analysis or multivariate analysis. However, the difference in serum CA-125 levels measured at the end of primary treatment between these two groups was statistically significant. In addition, this difference was still significant in stratified analysis and multivariate Cox regression analysis. These results indicated that posttreatment CA-125 level was an independent factor associated with the risk of disease recurrence. Furthermore, when we categorized patients into two arms by posttreatment CA-125 level at a cutoff of 10 U/ml, the median PFS was longer in patients who had a lower CA-125 level at the end of primary treatment compared with those with a posttreatment CA-125 level > 10 U/ml. This finding was consistent with previous studies [12, 13].

Even though there was a prospective study showed that measurement of CA-125 levels for the early detection of disease relapse did not improve outcomes in recurrent OC patients [14], the result of this trial was limited by the lack of new therapy (such as targeted therapy) and the lack of treatment of recurrent disease (such as the use of secondary cytoreductive surgery). With the development of targeted therapy (such as poly ADP-ribose polymerase inhibitor) which is a promising breakthrough in OC treatment, the role of monitoring posttreatment CA-125 level in prognosis value of epithelial OC is still worthy to be further investigated and discussed. However, in our experience from clinical practice and research over these 15 years, the reference value of serum CA-125 in predicting and monitoring disease progression should not be set at the same level in screening and diagnosing OC. In our present and previous studies, the suggested cutoff value was consistently at 10 U/ml. However, more high-quality and international studies are required to define the clinically significant cutoff value.

This study was a retrospective study from a preexisting cohort. Every patient had been counseled by an experienced gynecologic oncologist (the surgeon) prior to surgery treatment. Patients who were unlikely to be optimally cytoreduced were candidates treated by NACT with IDS. The result showed that there were more patients receiving NACT therapy prior to surgery than those directly undergoing PDS. In the univariate analysis, the proportion of patients with NACT was significantly larger in the recurrent group than that in the control group. However, the further subgroup analysis and Cox regression multivariate analysis showed no significant difference in PFS between the PDS and IDS arms. This result was consistent with the previous studies [15]. However, this result could not deny that NACT would improve the clinical response in advanced-stages cases and increase the probability of optimal cytoreduction at IDS [16]. Currently, the decision to identify patients who might benefit from NACT were made based on clinical factors by the surgeon. More reliable studies are needed to explore objective evaluation system to determine the reasonable application of NACT.

In conclusion, this study showed that a lower serum CA-125 level after primary treatment, as compared with a higher level, was a potentially good prognostic factor with regard to progression-free survival among women with advanced HGSOC. A new cutoff value of serum CA-125 level in monitoring disease progression might provide a substantial benefit in indication for postremission treatment such as maintenance target therapy, which is worthy of further investigation.

## Data Availability

Not applicable.
